# Long-term mesh complications and reoperation after laparoscopic mesh sacrohysteropexy: a cross-sectional study

**DOI:** 10.1007/s00192-020-04396-0

**Published:** 2020-07-03

**Authors:** Matthew L. Izett-Kay, Dana Aldabeeb, Anthony S. Kupelian, Rufus Cartwright, Alfred S. Cutner, Simon Jackson, Natalia Price, Arvind Vashisht

**Affiliations:** 1 Urogynaecology and Pelvic Floor Unit, University College London Hospitals, Clinic 2, Lower Ground Floor, EGA Wing, 235 Euston Road, London, NW12BU UK; 2 UCL EGA Institute for Women’s Health, University College London, Medical School Building, 74 Huntley Street, London, WC1E 6AU UK; 3 Department of Urogynaecology, John Radcliffe Hospital, Oxford University Hospitals, Headley Way, Oxford, Headington OX3 9DU UK

**Keywords:** Laparoscopy, Pelvic organ prolapse, Reoperation, Surgical mesh, Uterine prolapse

## Abstract

**Introduction and hypothesis:**

The paucity of long-term safety and efficacy data to support laparoscopic mesh sacrohysteropexy is noteworthy given concerns about the use of polypropylene mesh in pelvic floor surgery. This study is aimed at determining the incidence of mesh-associated complications and reoperation following this procedure.

**Methods:**

This was a cross-sectional postal questionnaire study of women who underwent laparoscopic mesh sacrohysteropexy between 2010 and 2018. Potential participants were identified from surgical databases of five surgeons at two tertiary urogynaecology centres in the UK. The primary outcome was patient-reported mesh complication requiring removal of hysteropexy mesh. Secondary outcomes included other mesh-associated complications, reoperation rates and Patient Global Impression of Improvement (PGI-I) in prolapse symptoms. Descriptive statistics and Kaplan–Meier survival analyses were used.

**Results:**

Of 1,766 eligible participants, 1,121 women responded (response proportion 63.5%), at a median follow-up of 46 months. The incidence of mesh complications requiring removal of hysteropexy mesh was 0.4% (4 out of 1,121). The rate of chronic pain service use was 1.8%, and newly diagnosed systemic autoimmune disorders was 5.8%. The rate of reoperation for apical prolapse was 3.7%, and for any form of pelvic organ prolapse it was 13.6%. For PGI-I, 81.4% of patients were “much better” or “very much better”.

**Conclusions:**

Laparoscopic mesh sacrohysteropexy has a low incidence of reoperation for mesh complications and apical prolapse, and a high rate of patient-reported improvement in prolapse symptoms. With appropriate clinical governance measures, the procedure offers an alternative to vaginal hysterectomy with apical suspension. However, long-term comparative studies are still required.

**Electronic supplementary material:**

The online version of this article (10.1007/s00192-020-04396-0) contains supplementary material, which is available to authorized users

## Introduction

A recent systematic review has highlighted a lack of high-quality data supporting mesh-augmented uterine-preserving surgical approaches for the treatment of uterine prolapse [1]. In the UK, the preferred procedure for the treatment of this highly prevalent condition is a vaginal hysterectomy (VH) and apical suspension procedure, with or without concomitant colporrhaphy [2, 3]. However, this approach is limited by a high risk of recurrent vault prolapse, rates of reoperation for prolapse are between 4.6% and 18% [4, 5]. More importantly, a significant proportion of women would prefer uterine preservation if given the option [6]. These two factors may explain the growing use of uterine-sparing techniques [7].

Laparoscopic mesh sacrohysteropexy is one such uterine-sparing procedure and involves re-suspending the uterus with polypropylene mesh anchored to the sacral promontory. Techniques are still evolving, but as undertaken in this study, the mesh was passed through two windows in the broad ligaments and anchored as a loop around the cervix. We have previously described this technique, as illustrated in Figure ESM 1 [8]. Reported advantages of laparoscopic sacrohysteropexy include lower blood loss and postoperative pain, longer vaginal length, and higher apical suspension compared with hysterectomy [1]. Our previously reported short-term data showed high rates of symptom relief and low complication rates at 3-month follow-up in over 500 women following this procedure [9]. Additionally, we reported longer term outcomes of 110 patients at an average of 2.6 years postoperatively, corroborating the findings of high satisfaction and low rates of reoperation for pelvic organ prolapse (POP) [10].

The paucity of quality evidence for such procedures is significant given the controversies surrounding implantation of pelvic mesh. Transvaginal mesh for prolapse has been recognised as having high rates of complications and there remain concerns amongst patients around transvaginal mesh use for stress urinary incontinence (SUI) [11, 12]. Uterine preservation may confer the benefit of a lower risk of mesh erosion than that seen in other abdominal approaches to prolapse. Following subtotal hysterectomy and cervicopexy, the reported mesh exposure rate is between 4.3% and 10.5% [13]. There have been multiple national and international reviews of mesh and in some countries pelvic mesh remains partially or completely restricted from use [14–18]. Although mesh complications may be asymptomatic, they are often associated with chronic pain, leading to devastating impacts on quality of life [19]. Despite quality evidence refuting an association between systemic autoimmune disorders and mesh, some patients continue to worry about a possible link [20]. Regulators and reports into mesh have repeatedly identified a need for high-quality long-term cohort studies of pelvic mesh surgery [21, 22]. On the basis of our clinical experience over the last decade we hypothesised the rate of these mesh-associated complications to be very low.

The aim of this study was to determine the rates of reoperation and mesh-associated complications in women who had undergone laparoscopic mesh sacrohysteropexy.

## Materials and methods

We conducted a multicentre cross-sectional questionnaire study of women who underwent laparoscopic mesh sacrohysteropexy between February 2007 and September 2018, a timeframe from when the procedure was routinely offered until the start of this study. Potential participants were identified from the operating databases of five consultant surgeons based at two tertiary urogynaecology centres in the UK (University College London Hospitals, London and Oxford University Hospitals, Oxford), using operating procedure codes (OPCS) Y75.2 (laparoscopic approach to the abdominal cavity) or T43.9 (unspecified diagnostic endoscopic examination of the peritoneum) in combination with Q54.1 (suspension of the uterus NEC), Q54.4 (suspension of the uterus using mesh) or Q54.5 (sacrohysteropexy).

We included all English-speaking women over the age of 18 who underwent laparoscopic mesh sacrohysteropexy at one of our participating centres utilising the technique previously described, with steps illustrated in Figure ESM 1 [8]. This involves the use of a bifurcated polypropylene mesh wrapped around the cervix through broad ligament windows and secured anteriorly with non-absorbable sutures (Ethibond Excel™; Ethicon) that is then secured to the sacral promontory with a helical fastener (Protack™; United States Surgical, Tyco Healthcare, Norwalk, CT, USA). According to local procurement policies the mesh used was either PRO-Lite™ (Atrium Medical Corporation, Hudson, NH, USA) or Prolene™ mesh (Ethicon, Somerville, NJ, USA). The mesh used for individual participants was not available.

We excluded any patient who underwent a previous or subsequent trans-vaginal mesh-augmented prolapse operation, or concurrent mesh rectopexy and did not contact or include patients who were identified as deceased on our hospital databases.

The questionnaire used was designed to appropriately capture the outcome measures defined below and is contained in Appendix 1. Owing to the rare nature of many of the study’s main outcomes, it was not possible to formally validate or test the reliability of the questionnaire prior to commencing the study. The questionnaire items were developed by the senior authors and then piloted at one site. This involved completion by women fulfilling our study inclusion criteria, who provided written or verbal feedback with respect to question comprehensibility. Potential participants were then contacted by post and able to respond by post in a prepaid envelope, or request a telephone questionnaire. Alternatively, they could submit responses using the REDCap electronic data capture tools hosted University College London [23, 24]. Research Electronic Data Capture (REDCap) is a secure, web-based software platform designed to support data capture for research studies, providing:

1. An intuitive interface for validated data capture
2. Audit trails for tracking data manipulation and export procedures
3. Automated export procedures for seamless data downloads to common statistical packages
4. Procedures for data integration and interoperability with external sources

Telephone interviews for the questionnaire were carried out according to a telephone script following verbal consent. Potential participants who had not responded to the first postal contact within 8 weeks were sent a second questionnaire.

Our study protocol was registered with the UK’s Health Research Authority (HRA) and received a favourable research ethics committee (REC) opinion from the London City & East REC on 11/05/2018 (reference 18/LO/0637), and favourable HRA approval. Participants gave consent to allow the study team to contact clinicians who managed any mesh complications, to obtain further details.

The primary outcome for our study was patient-reported mesh complications requiring the removal of the hysteropexy mesh. Patients were also asked the nature and timing of symptoms that led to the diagnosis of this mesh-associated complication. Secondary outcomes included the use or expectant use of chronic pain services, and the new diagnosis of a systemic autoimmune disorder (see Appendix 1). Further secondary outcomes included subsequent reoperation for POP and type of procedure, reoperation for SUI, Patient Global Impression of Improvement in prolapse symptoms (PGI-I prolapse) and the “Friends and Family test”, asking whether participants would recommend the surgery if undergoing treatment for the same condition. All reported results come from the patient-reported data contained within the questionnaire responses, with the exception of the case details of those patients who reported reoperation for a mesh complication. In these nine women, case notes were obtained where available and with consent, for clarification of the nature of their reported mesh-associated complication.

Available data were analysed using descriptive statistics, with frequencies expressed as percentages. Survival analyses for mesh excision and reoperation for POP as the failure variables were undertaken using the Kaplan–Meier method. These analyses were performed using Stata/SE 15® (StataCorp, College Station, TX, USA).

## Results

**Fig. 1:**
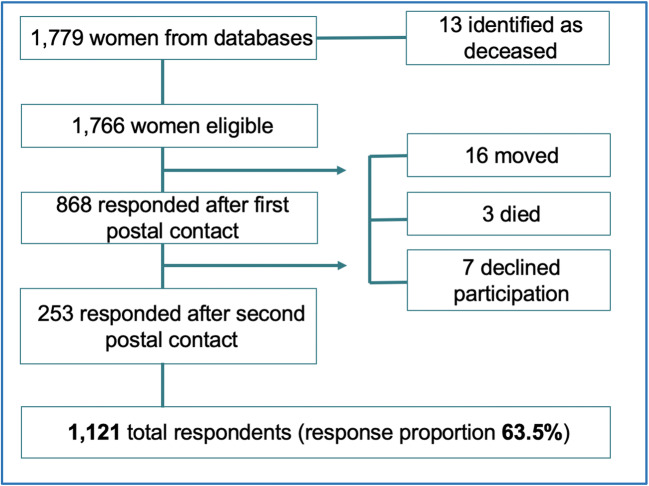
Flow chart of participant recruitment

**Fig. 2:**
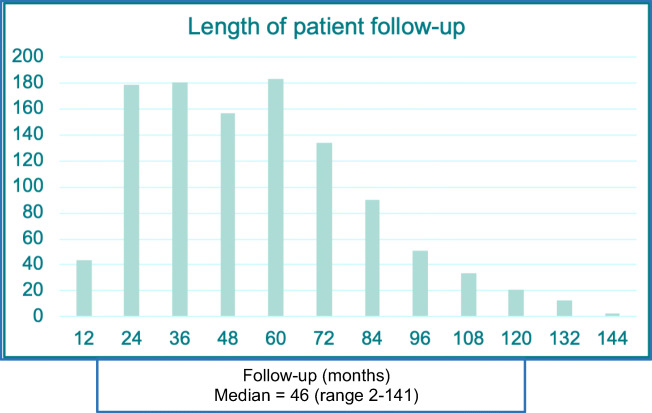
Length of patient follow-up

We identified 1,766 potential participants, and following two rounds of postal contact, 1,121 women responded (response proportion 63.5%), as shown in Fig. 1. The median length of follow-up from index hysteropexy was 46 months (range 2–141 months), shown in Fig. 2. The average age of participants at the time of surgery was 58 years (range 24–86 years), other patient demographic details were unavailable owing to the nature of the surgical databases.

### Primary outcome

**Fig. 3:**
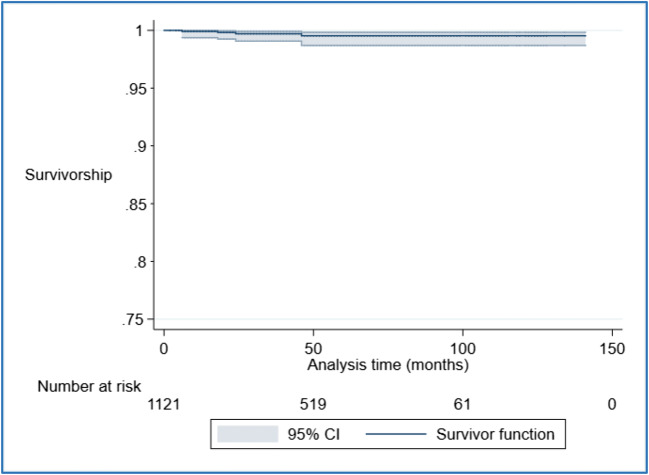
Kaplan–Meier survival analysis with mesh removal surgery as the failure variable

The incidence of patient-reported mesh complications requiring removal of hysteropexy mesh, confirmed by case note review, was 0.4% (4 out of 1,121). This equated to 0.86 mesh removal operations per 1,000-person years of follow-up. Figure 3 illustrates the Kaplan–Meier survival analysis, with patient-reported mesh complication requiring removal of the hysteropexy mesh as the failure variable. All reoperations for patient-reported mesh complications were undertaken within 4 years of the sacrohysteropexy. Details of the four cases in which participants reported mesh excision surgery are shown in Table 1.

**Table 1:** Case details of patient-reported mesh complications

	Age at surgery	Time from sacrohysteropexy to mesh removal surgery (months)	Case details
**1**	54	–	Year of surgery: 2008
			No hospital notes available for further analysis
**2**	68	46	Year of surgery: 2013
			Implant: unknown
			Grade of surgeon: consultant Sacrohysteropexy details: unremarkable
			Indication for mesh removal: acute small bowel obstruction
			Preoperative imaging: CT—small bowel obstruction
			Conservative management: N/A
			Operation and approach: midline laparotomy, partial small bowel resection, partial excision of hysteropexy mesh with re-peritonealisation
			Intraoperative findings: suspected SBO due to mesh, small 1–2 cm exposure of mesh at broad ligament—excised, and mesh end peritonealised, additional non-tensioned peritonealised mesh at sacral aspect of implant was excised and peritonealised
			Recovery: chronic abdominal pain, noted adhesions—managed conservatively, ventral hernia repaired with mesh
			Clavien–Dindo: IIIb
**3**	64	18	Year of surgery: 2014
			Implant: Prolite mesh, Atrium Medical, 5 cm × 30.5 cm
			Grade of surgeon: SST
			Sacrohysteropexy details: diverticular disease noted
			Indication for mesh removal: lower abdominal and back pain, recurrent prolapse
			Preoperative imaging: none
			Conservative management: plication of mesh
			Mesh removal surgery: laparoscopic complete resection of mesh and ProTack, laparoscopically assisted vaginal hysterectomy, anterior colporrhaphy
			Mesh removal surgery findings: elongated cervix at −1, cystocele +2
			Recovery: uncomplicated, seen at 3/12 and discharged with no issues
			Clavien–Dindo: IIIb
**4**	40	24	Year of surgery: 2016
			Implant: Prolene, Ethicon, 15 cm × 15 cm
			Grade of surgeon: subspecialty trainee
			Sacrohysteropexy details: unremarkable
			Indication for mesh removal: abdominal and vaginal pain, dyspareunia
			Conservative management: PFMT, paracetamol, amitriptyline
			Operation and approach: total laparoscopic hysterectomy with complete removal of mesh and ProTack
			Intraoperative findings: unremarkable
			Recovery: ongoing vaginal “soreness”, discharged at 3 months
			Clavien–Dindo: IIIb

Five patients reporting sacrohysteropexy mesh removal surgery were not included in the reporting of our primary outcome. One participant had undergone a concurrent synthetic mid-urethral sling for urinary incontinence at the time of sacrohysteropexy. She presented 18 months postoperatively with vaginal pain and dyspareunia and was found to have a small exposure of sub-urethral mesh. Partial excision of the sub-urethral portion of the tape from a vaginal approach was undertaken and the hysteropexy mesh was left in situ. She was followed up for 3 months and subsequently discharged. A second participant complained of abdominal pain within 4 weeks of surgery and was found to have haematometra. She underwent laparoscopic subtotal hysterectomy; the mesh was left in situ and used to undertake a stump cervicopexy. She was subsequently discharged from follow-up. The final 3 patients reporting reoperation for a mesh-associated complication had undergone reoperation for recurrent prolapse, 2 requiring mesh plication and the third opting for a vaginal hysterectomy.

### Secondary outcomes

With respect to symptoms leading to removal of mesh, 2 participants reported pain and 2 reported bladder symptoms, as detailed in Table 2. Three women reported noticing the symptoms associated with the mesh complication as having developed within 12 months of their hysteropexy; details of these patient-reported data are shown in Table 2.

**Table 2:** Patient- reported events leading to mesh complication

Parameter	Data,* n* (%)
Reason for mesh removal	
Asymptomatic	–
Pain on examination	–
Pain during sex	–
Pain during physical/daily activities	1 (25%)
Pain unrelated to above	1 (25%)
Vaginal discharge	–
Bladder symptoms	2 (50%)
Bowel symptoms	–
Timeframe from operation to symptoms of mesh complication	
< 48 h	–
49 h to 2 months	1 (25%)
3 months to 12 months	2 (50%)
> 12 months	1 (25%)

For the other patient-reported mesh-associated complications included within this study, 1.8% of the study participants (20 out of 1,121) reported that they had previously been or were awaiting referral to chronic pain services for pain specifically attributed to the mesh. With respect to systemic autoimmune diseases, 5.8% (65 out of 1,121) of participants reported a new diagnosis of such a condition subsequent to their laparoscopic mesh sacrohysteropexy.

The risk of subsequent reoperation for POP was 13.6% (152 out of 1,121), and for SUI it was 2.3% (26 out of 1121), with details shown in Table 3. With respect to PGI-I prolapse, 81.4% of participants (912 out of 1,121) reported their symptoms to be “very much better” or “much better”, and 82.2% (921 out of 1,121) would recommend the procedure to a friend or family member with the same condition.

**Table 3:** Subsequent procedures for pelvic organ prolapse (*POP*) and stress urinary incontinence (*SUI*)

Parameter	Data,* n* (%)
Subsequent POP procedure (*N* = 152)	
Apical procedure	41 (3.7%)
Hysterectomy	9 (0.8%)
Colporrhaphy	102 (9.1%)
Subsequent SUI procedure (*N* = 26)	
Synthetic mid urethral sling	13 (1.2%)
Mid urethral fascial sling	1 (0.1%)
Periurethral bulking	3 (0.4%)
Colposuspension	5 (0.5%)
Unspecified	4 (0.4%)

## Discussion

### Main findings

We report a low incidence of patient-reported mesh complications requiring mesh removal surgery confirmed by case note review, of 0.4% at a median follow-up of nearly 4 years, from a cohort of 1,121 women who underwent laparoscopic mesh sacrohysteropexy. Notably, there were no reported cases of vaginal mesh erosion.

This is the largest reported study of women who have undergone mesh-augmented uterine-preserving prolapse surgery, and the incidence of reoperation for a mesh-associated complication compares favourably with other gynaecological uses of mesh. For comparison, the risk of reoperation for a mesh complication following two of the most common such procedures, sacrocolpopexy and the synthetic mid-urethral sling, are 5% and 2.4% respectively [25, 26]. According to the Cochrane review by Maher et al. [27] the risk of reoperation for mesh exposure following placement of transvaginal mesh for prolapse is 8%, and in the PROSPECT trial, the largest randomised trial of transvaginal mesh, 4% of women required reoperation for a mesh complication [12, 27].

Comparison of our findings with the literature for sacrohysteropexy is difficult owing to the heterogeneity of reporting mesh complications. Long-term cohort studies have reported mesh erosion rates of 4% following robotics-assisted sacrohysteropexy, and 5% following open sacrohysteropexy, with no details regarding reoperation [28, 29]. A variety of surgical techniques exist for sacrohysteropexy, with no clear evidence to support one particular approach over another. Our technique differs from others reported in the literature in that there is limited dissection on the vagina, and mesh arms are sutured to the anterior cervix. It may be that the avoidance of mesh placement on the vagina explains the absence of vaginal mesh erosions at an average follow-up of nearly 4 years in contrast to the complications seen in these other series. Comparing mesh sacrohysteropexy and vaginal hysterectomy in a systematic review, Meriwether et al. did not report any cases of mesh erosion or reoperation following abdominal mesh sacrohysteropexy [1]. Most of the studies included in this part of the review either failed to report these outcomes, or they were only seen in the comparator groups undergoing hysterectomy and concurrent sacrocolpopexy, where the risk of reoperation for a mesh complication was between 2 and 3%.

With respect to the other mesh-associated complications, the risk of utilising chronic pain services in our study appears relatively low; following vaginal hysterectomy, the risk of chronic pain may be as high as 25% [30]. Our finding of a 5.8% risk of subsequent diagnosis of a systemic autoimmune disorder is higher than the 2.8% risk reported by the largest and most methodologically robust available study looking at the association between gynaecological mesh and autoimmune disease [20]. However, in that matched cohort study, over 40% of women undergoing mesh-augmented POP surgery had pre-existing diagnoses of autoimmune conditions, and were not included in the analysis. We were unable to account for pre-existing autoimmune disease in our participants to allow for exclusion from our analysis, and therefore this finding should be interpreted with caution.

It is noteworthy that in our study only 3.7% of our patients underwent a subsequent apical prolapse procedure, compared with the 6–8% risk reported following vaginal hysterectomy [31]. It is uncertain if this finding supports the role of mesh augmentation for repair of apical prolapse; the issue remains controversial. Reoperation rates for prolapse of any compartment in our study are comparable with the overall reoperation rate for POP following VH, which was estimated to be 11% at 5 years in a recent large Danish registry study [32]. It is significantly lower than reoperation rates following suture hysteropexy, which was reported to be 30% in the same study. The 9% risk of subsequent colporrhaphy in our study may reflect surgical practice within participating centres. Concurrent anterior and posterior compartment repair for prolapse above the hymenal ring is generally avoided, as evidence suggests that such prolapse might be less likely to be symptomatic, and could be considered normal [33]. The authors acknowledge that this may lead to higher rates of colporrhaphy at a later date; indeed, our own reoperation rates in this study are higher than those reported in our previous cohorts, likely a feature of the longer follow-up [9, 10]. Reoperation for SUI is a recognised risk of POP surgery; the incidence of this in our study is comparable to the 2% risk quoted in large national studies [34]. The PGI-I prolapse has been validated as a measure for POP surgery, and we report high rates of improvement in prolapse symptoms [35].

### Significance and implications

The findings from our study are important given the large cohort, long-term follow-up, and importantly, the patient-reported nature of our outcomes. Despite well-publicised controversies surrounding the use of mesh in pelvic floor surgery, the majority of patients would recommend laparoscopic mesh sacrohysteropexy to friends or family. This, in combination with a low risk of reoperation for mesh complications and low rates of chronic pain service use, should provide reassurance to both clinicians and women considering the procedure, as well as regulatory bodies.

The role of mesh-augmented prolapse surgery, specifically sacrohysteropexy, deserves ongoing scrutiny. Although the hierarchy of evidence-based medicine would favour the need for large, multicentre prospective studies, these are notoriously difficult for surgical interventions. Additionally, the controversies surrounding mesh in pelvic floor surgery are likely to make such studies difficult to undertake. Although large database studies would be helpful, researchers often depend on data from clinicians or hospital coders, who have their own issues with respect to accuracy. Therefore, such studies are valuable in providing a pragmatic evidence base for women considering surgery for POP, as well as clinicians and regulators.

### Strengths and limitations

The principle strengths of our study include the long-term follow-up of a large number of participants, operated on by several surgeons at different institutions using a standardised follow-up in the form of a questionnaire. The value of patient-reported data cannot be understated given the current climate surrounding mesh use, with anecdotal concerns about a potential breakdown in trust between patients and their clinicians [36].

There are methodological limitations inherent within our study design, such as a lack of a comparison group, the use of patient-reported data without routine clinical or case note review to confirm the reported outcomes, and a paucity of demographic data to allow for regression analysis. Owing to the rare nature of our primary outcome, the use of a validated patient-reported outcome measure questionnaire was not possible. Study participants underwent their procedures in centres with specific expertise in laparoscopic pelvic floor surgery, and therefore the research setting and findings may not be applicable to other centres. As with all questionnaire studies, results are affected by recall and response bias of participants. Participants may well not recall undergoing a subsequent surgical procedure or the nature of such surgery. Consideration of these biases also applies to the interpreting the use of chronic pain services, and subsequent diagnosis of a systemic autoimmune disorders. Finally, owing to the lack of demographic data, we cannot comment on how representative our participants are of the whole cohort of potential participants. Those greatly troubled by mesh complications may have opted not to participate or, conversely, were keenest to respond. Similarly, our subjective outcomes of PGI-I prolapse and the “friends and family” test may be adversely influenced by mesh concerns and media coverage.

## Conclusion

This study provides a large, patient-reported dataset offering a pragmatic insight with respect to the wider context of other forms of available evidence on the safety and efficacy of mesh-augmented sacrohysteropexy. In our opinion, the low risk of reoperation for a mesh-associated complication provides reassurance that laparoscopic mesh sacrohysteropexy can continue to be offered with appropriate decision-making processes, consent, clinician training and audit.

## Electronic supplementary material

ESM 1 (DOCX 1522 kb)

ESM 2 (DOCX 41 kb)
