# Supplementary material for: Long-term mesh complications and reoperation after laparoscopic mesh sacrohysteropexy: a cross-sectional study
Source: Int Urogynecol J. 2020 Jul 3;31(12):2595–602. doi: 10.1007/s00192-020-04396-0 (PMC7679361; doi:10.1007/s00192-020-04396-0)
Supplement: Supplementary file 1 — (DOCX 1522 kb) [file 192_2020_4396_MOESM1_ESM.docx]

Figure ESM1. Surgical technique. Key steps of laparoscopic mesh sacrohysteropexy.


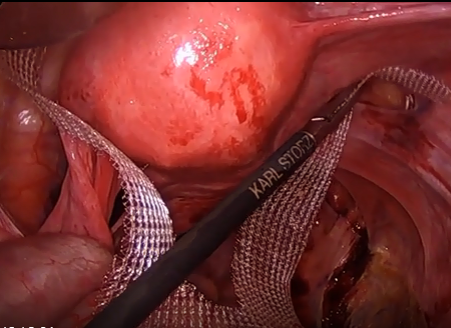

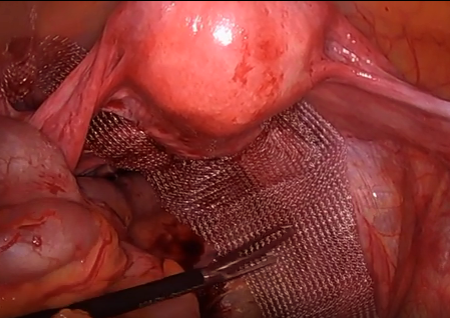

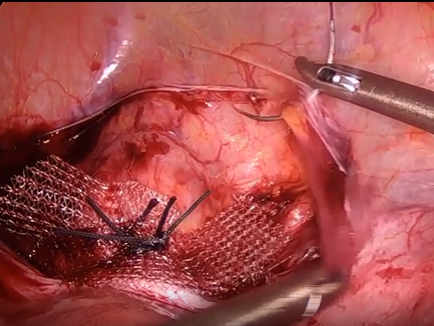


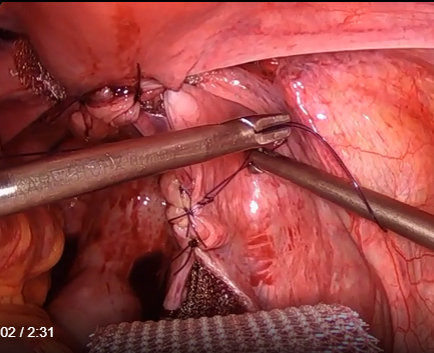


**4**
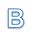

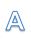


**3**
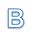

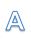


**2**
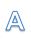


**1**

**Surgical technique:**

1) Mesh graft cut to provide two bifurcated arms to secure at level of cervico-uterine junction.

2) Mesh arms passed through bilateral windows made in broad ligament.

3) Arms of mesh secured to anterior cervix and then reperitonealised.

4) Body of mesh graft reperitonealised, secured to sacral promontory and finally the sacral promontory is reperitonealised.
