# Supplementary material for: Long-term mesh complications and reoperation after laparoscopic mesh sacrohysteropexy: a cross-sectional study
Source: Int Urogynecol J. 2020 Jul 3;31(12):2595–602. doi: 10.1007/s00192-020-04396-0 (PMC7679361; doi:10.1007/s00192-020-04396-0)
Supplement: Supplementary file 2 — (DOCX 41 kb) [file 192_2020_4396_MOESM2_ESM.docx]

**Confidential Patient Questionnaire**

Please read the attached patient information sheet before completing. By returning this questionnaire you are consenting to participation within the study as detailed within the attached information sheet. For convenience, this form can be completed ONLINE with the link provided to you.

Please answer the following questions **in reference to your keyhole prolapse operation**:

|  |  |  |  |  |  |
| --- | --- | --- | --- | --- | --- |

Today’s date (DD/MM/YY):

1. **Following your keyhole prolapse surgery, have you required an operation to remove some or all of the mesh used to lift up your womb?**

Yes No

If **Yes**, proceed to **Question 2**

If **No**, proceed to **Question 6**

1. **Which of the following best describes the operation to remove the mesh ?**

***(Please only select one)***

Removal of mesh from the vagina

Removal of mesh from the bowel

Removal of mesh from the bladder

Removal of mesh due to chronic pain

1. **How long after your keyhole prolapse surgery did you first notice the problem caused by the mesh?**

Less than 48 hrs 48 hrs – 2 months

Between 2 and 12 months more than 12 months

1. **Please provide the following details about the operation you had to deal with the mesh complications (*leave blank if unsure*):**

Name of Hospital: __________________________________________________

Name of Consultant: ________________________________________________

Year operation performed: ___________________________________________

Continued overleaf……………….

1. **Which of the following best describes the problems caused by your mesh complication? *(please only select one)***

No symptoms

Pain on physical examination

Pain during sexual intercourse

Pain during physical / daily activities

Pain that is not related to the above

Vaginal discharge

Bladder symptoms

Bowel symptoms

1. **Following your keyhole prolapse surgery, have you required another operation to treat further prolapse?**

Yes No

**If yes,** select the option that best describes the operation you have had:

Another operation to lift the up womb

Removal of the womb, also known as hysterectomy

An operation for prolapse of the vaginal walls

1. **Following your keyhole prolapse surgery, have you required a procedure to treat NEW leaking of urine when you cough, sneeze, run and/or strain (stress incontinence)**?

Yes No

**If yes,** please select the best description of the operation you have had (leave blank if unsure):

A mesh sling, also known as a TVT or vaginal tape

A fascial sling, using tissue taken from your abdomen

The injection of a ‘bulking agent’ into your water pipe

A colposuspension, using stitches to support the bladder

Continued overleaf……………….

1. **Following your keyhole prolapse surgery, have you been newly diagnosed with any of the following medical conditions? *(select all that apply)***

Underactive/overactive thyroid

Autoimmune/Pernicious Anaemia

(B12 deficient anaemia)

Autoimmune thrombocytopaenic purpura (low platelets)

Guillain-Barre Syndrome

Goodpasture Syndrome

Vasculitis

Coeliac Disease

Pemphigous Vulgaris

Polymyositis

Multiple Sclerosis

Myaesthenia Gravis

Rheumatoid Arthritis

Fibromyalgia

Systemic Lupus Erythematosus

Motor Neurone Disease

Dermatomyositis

Ankylosing Spondylitis

Sjogren’s Syndrome

Systemic Sclerosis

1. **Following your keyhole prolapse surgery, have you been referred to or are you currently under the care of pain specialist due to problems because of mesh?**

Yes No

1. **Would you recommend keyhole prolapse surgery to a friend?**

Yes No

1. **Overall, which of the following best describes your PROLAPSE symptoms (specifically feeling a lump, bulge or heaviness in the vagina) now, compared with before your surgery?**

Very much better

Much better

A little better

No change

A little worse

Much worse

Very much worse

Continued overleaf……………….

1. **Are there any comments of further information you would like to provide to the research team about the operation, your recovery and/or current symptoms with regards to general health and prolapse? *(There may be a delay in reviewing your response so please ensure to see your doctor if you are having any current issues)***

________________________________________________________________

________________________________________________________________

________________________________________________________________

1. **If you would like a copy of the final report, select one of the following:**

**Post: or, Email:** ____________________________________________

Thank you for taking the time to complete this questionnaire. Please return in the pre-addressed envelope.
